# Metagenomic data of soil microbial community in relation to basal stem rot disease

**DOI:** 10.1016/j.dib.2020.106030

**Published:** 2020-07-15

**Authors:** Racheal Khai Shyen Lo, Khim Phin Chong

**Affiliations:** a Biotechnology Programme, Faculty of Science and Natural Resources, Universiti Malaysia Sabah, 88400 Jalan UMS, Kota Kinabalu, Sabah, Malaysia; b FGV Chair of Sustainable Oil Palm Management, Faculty of Agriculture, Universiti Malaysia Sabah, Mile 10, Sg. Batang, 90000 Sandakan, Sabah, Malaysia

**Keywords:** 16S metagenomics, Soil microbial communities, Basal stem rot, Oil palm

## Abstract

The oil palm industry, especially in Indonesia and Malaysia is being threatened by Basal Stem Rot (BSR) disease caused by *Ganoderma boninense*. There is no conclusive remedy in handling this disease effectively. In this study, metagenomics analysis of soil were analyzed for a better understanding of the microbial diversity in relation to BSR disease. Study was conducted in three plantation sites of Sabah, Malaysia which incorporated different disease management and agronomic practices. The estates are located at Sandakan (Kam Cheong Plantation), Lahad Datu (FGV Ladang Sahabat) and Tawau (Warisan Gagah). Soil samples were collected from disease free, high and low BSR incidence plots. Illumina MiSeq metagenomic analysis using V3–V4 region of 16S rRNA gene was employed to study the microbial diversity. Bacteria (97.4%) and Archaea (0.2%) were found majority in kingdom taxonomy level. The most abundant phyla were *Proteobacteria, Acidobacteria, Actinobacteria*, and *Verrucomicrobia*. Higher alpha diversity of all species was observed among all tested soil from each estates. Beta analysis was analyzed using non phylogenetic UnifRac matrix and visualized using Principal Coordinates Analysis (PCoA). The tested soil samples in Kam Cheong Plantation were found to have similar bacterial communities. The data provided is useful as an indicator in developing biology controls against *Ganoderma boninense.*

Specifications Table

**Table:** 

Subject	Biology
Specific subject area	Metagenomics
Type of data	Figure
How data were acquired	NGS sequencing on Illumina MiSeq platform
Data format	Raw, analyzed
Parameters for data collection	llumina MiSeq metagenomic analysis using V3–V4 region of 16S rRNA gene was employed to study the microbial diversity.
Description of data collection	Oil palm soil with disease free, high and low basal stem rot disease were collected. DNA of the soil samples were extracted using PowerSoil® DNA Isolation Kit (MoBio Laboratory, CA, USA) and were submitted for next generation sequencing of 16s rRNA gene.
Data source location	City/Town: 1. Sandakan (Kam Cheong Plantation) 2. Lahad Datu (FGV Ladang Sahabat) 3. Tawau (Warisan Gagah) Region: Sabah Country: Malaysia Latitude and longitude (and GPS coordinates) for collected samples/data: 1. Kam Cheong Plantation (5°50′30.65"N, 117°48′48.92"E) 2. FGV Ladang Sahabat (5°9′55.76"N, 119°8′42.00"E) 3. Warisan Gagah (4°22′48.21"N, 118°9′47.96"E)
Data accessibility	Raw sequencing data are hosted in the public repository Discover Mendeley Data with direct URL to data: https://data.mendeley.com/datasets/5cxw9f5ngz/2

## Value of the data

• Basal stem rot caused by *Ganoderma boninense* remains the most important disease which cost billion of losses to Malaysia and Indonesia economy in a year. Managing the disease is one of the main agenda of two countries for industrial crop.
• These data gives insight to the microbial diversity of oil palm soil which involving disease free, high and low basal stem rot incidences.
• Soil microbial taxonomy can be used as a reference in developing biology controls against *Ganoderma boninense.*

## 1 Data description

**Figure 1:**
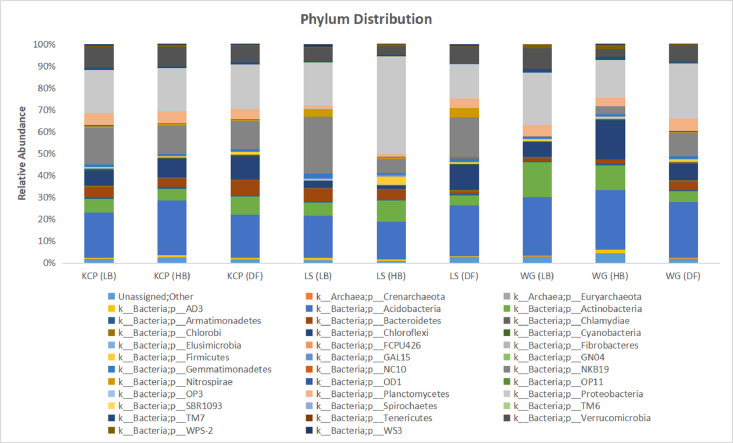
Phylum distribution of the tested soil samples in three estates in Sabah which incorporate different disease management and agronomic practices. Note: KCP = Kam Cheong Plantation, LS = Ladang Sahabat, WG = Warisan Gagah, LB = Low BSR, HB = High BSR, DF = Disease Free

**Figure 2:**
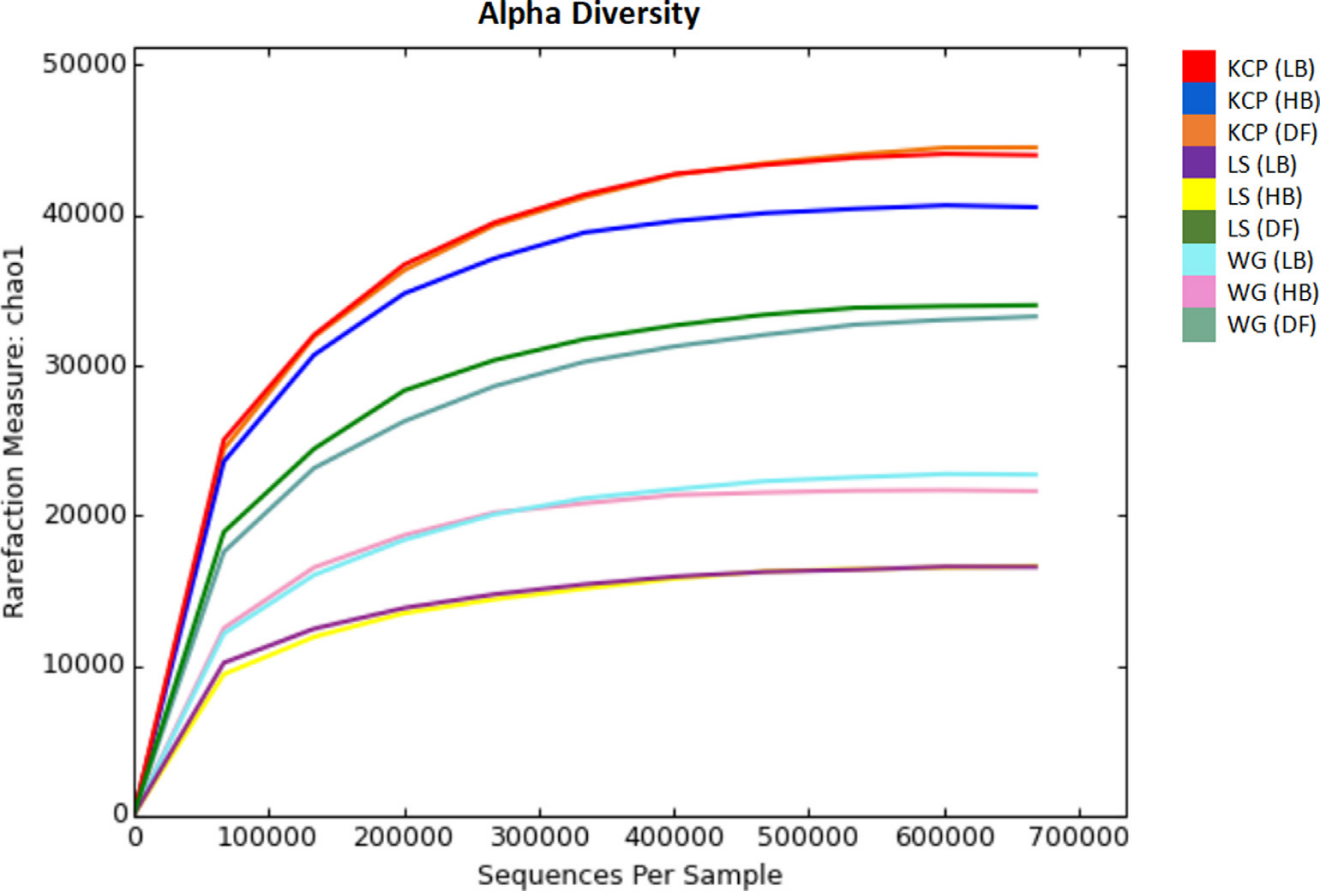
Chao1 species richness estimation in the soil samples of three estates in Sabah which incorporate different disease management and agronomic practices. Note: KCP = Kam Cheong Plantation, LS = Ladang Sahabat, WG = Warisan Gagah, LB = Low BSR, HB = High BSR, DF = Disease Free

**Figure 3:**
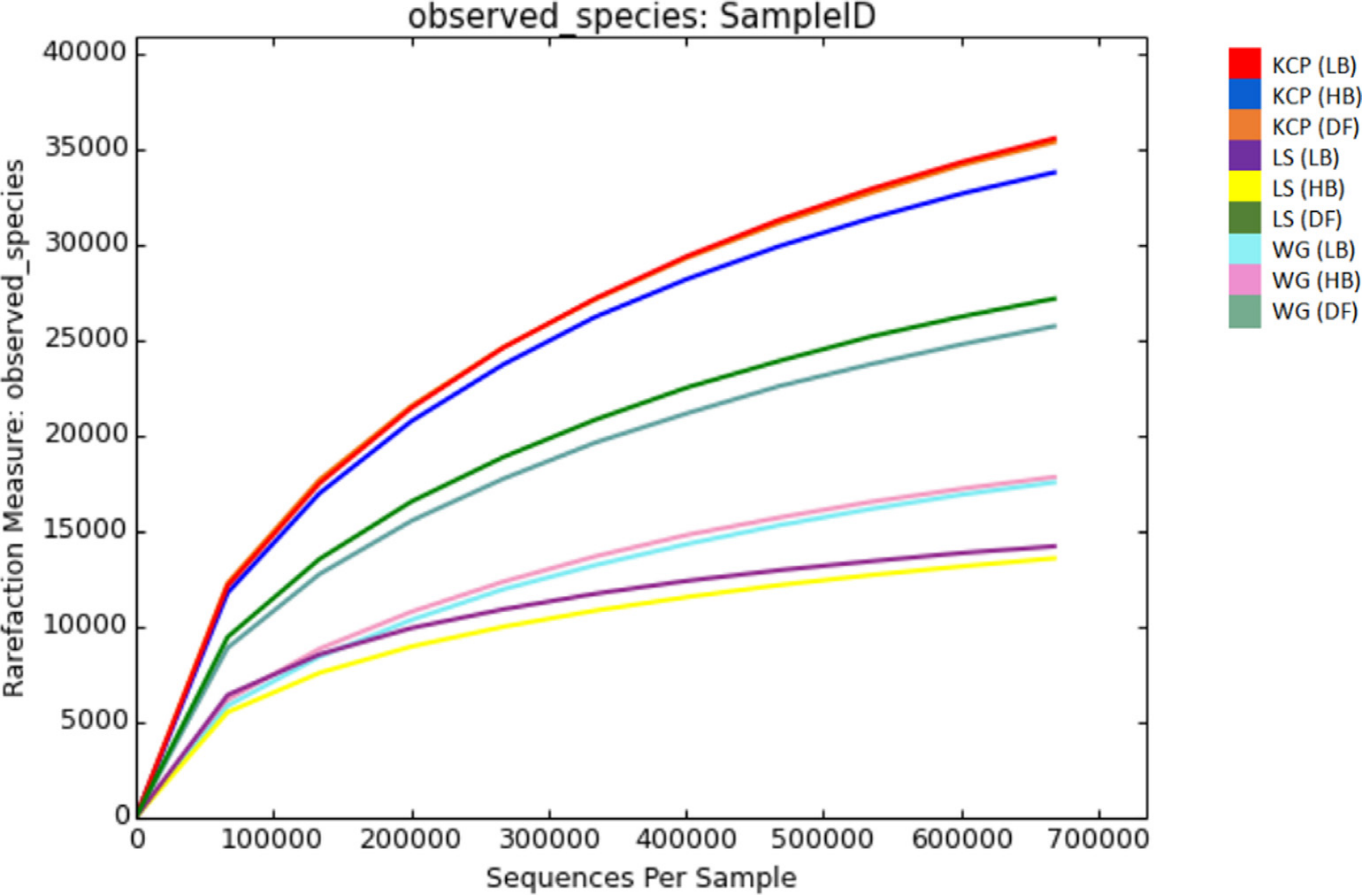
Number of Observed OTUs in the soil samples of three estates in Sabah which incorporate different disease management and agronomic practices. Note: KCP = Kam Cheong Plantation, LS = Ladang Sahabat, WG = Warisan Gagah, LB = Low BSR, HB = High BSR, DF = Disease Free

**Figure 4:**
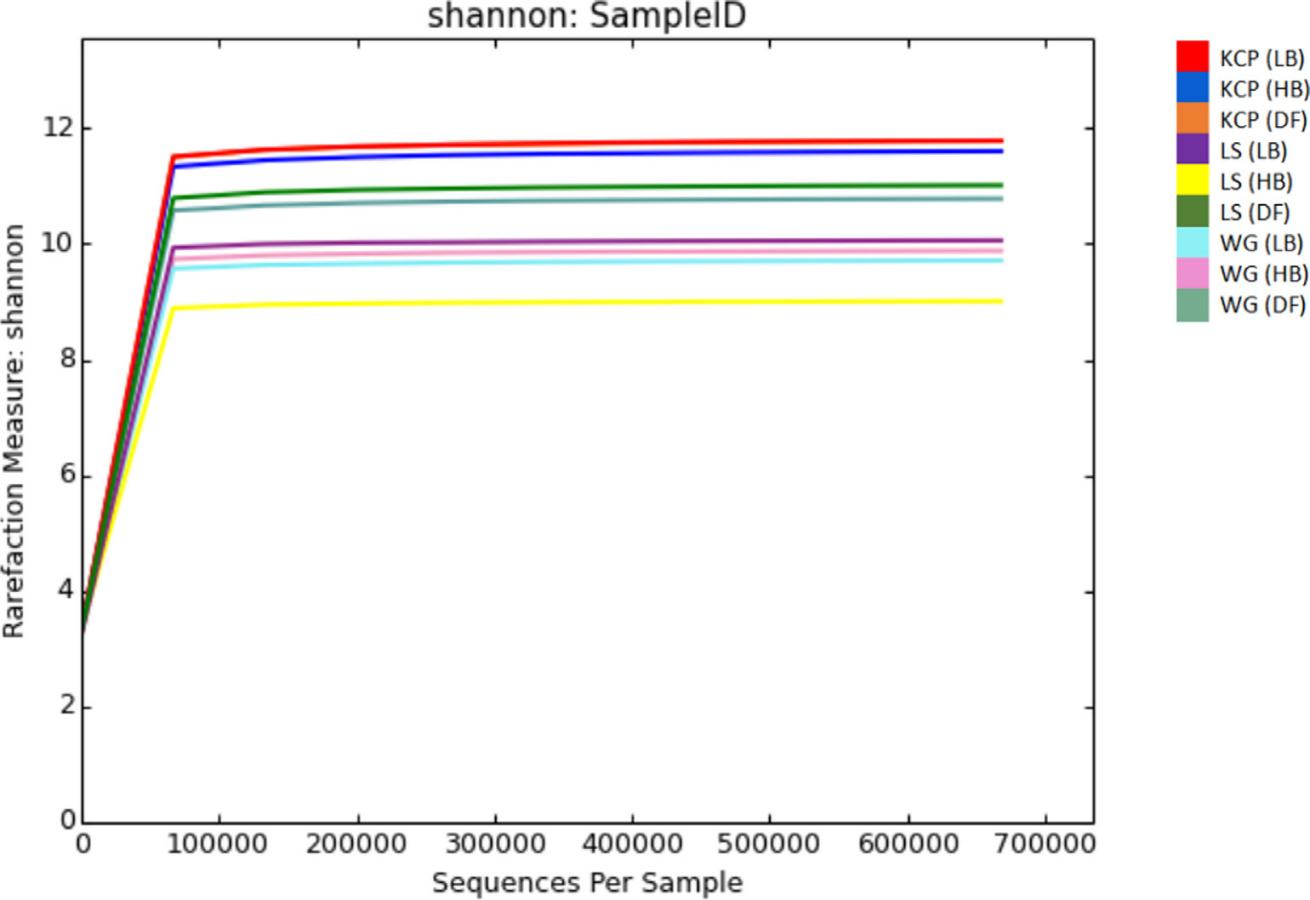
Shannon Diversity in the soil samples of three estates in Sabah which incorporate different disease management and agronomic practices. Note: KCP = Kam Cheong Plantation, LS = Ladang Sahabat, WG = Warisan Gagah, LB = Low BSR, HB = High BSR, DF = Disease Free

**Figure 5:**
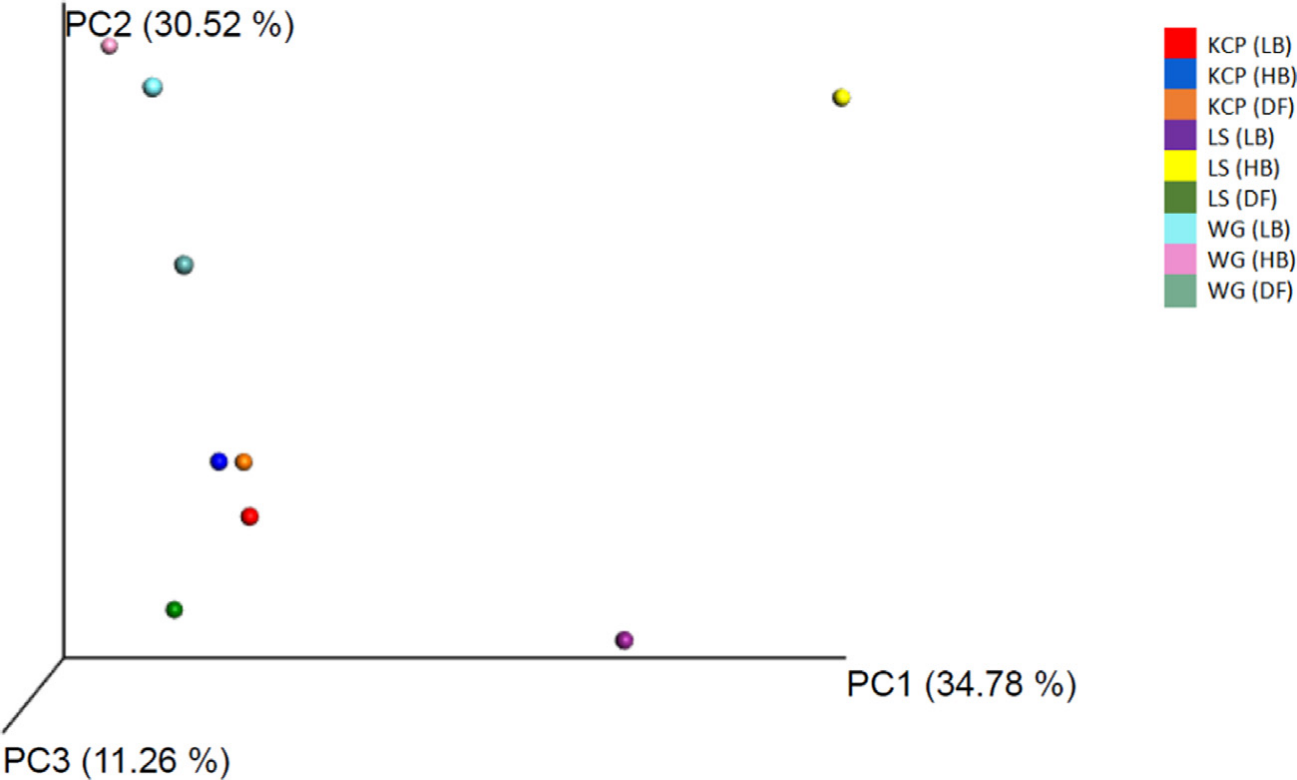
Principle coordinate analysis (PCoA) visualization based on weighted UniFrac Martix in three estates in Sabah which incorporate different disease management and agronomic practices. Note: KCP = Kam Cheong Plantation, LS = Ladang Sahabat, WG = Warisan Gagah, LB = Low BSR, HB = High BSR, DF = Disease Free

Oil palm plantation remains threatened by the Basal Stem Rot (BSR) disease caused by *Ganoderma boninense*. BSR infection can lead to palm death and yield losses [1]. To date, there is no solution in controlling this disease effectively. Soil contains enormous microbial communities which is important in regulating nutrient and biogeochemical cycles [2]. A true insight of soil microbial communities and compositions is necessary to develop new strategies in managing BSR. The data provided contributes to develop more comprehensive diagnosis and biological controls for BSR disease treatment. The dataset consists of raw paired-end sequencing data of 16S rDNA metagenomics where DNA was isolated from three different soil conditions (disease free, high and low BSR incidences) in three different oil palm estates which incorporate different disease management and agronomic practices. Kam Cheong Plantation applied organic acids, FGV Ladang Sahabat carried out sanitation and Warisan Gagah applied microbial products in managing BSR disease. The overall raw sequencing data contain 100,355 with a total average read count of 759,520 base pairs. Data file was deposited at the public respository Discover Mendeley Data (https://data.mendeley.com/datasets/5cxw9f5ngz/2). Bacteria (97.4%) and Archaea (0.2%) was found majority in the representative kingdoms taxonomy level. Information about the phylum distribution, alpha diversity (Chao1, observed OTUs, Shannon) and beta diversity (PCoA) is presented in Fig. 1 until Fig. 5 respectively. Data file related to the figures was deposited at the public respository Discover Mendeley Data (https://data.mendeley.com/datasets/5cxw9f5ngz/2). Soil with high BSR disease in Ladang Sahabat appeared to affect soil microbial communities and diversity the most compared to other estates. Presence of *Ganoderma* may has an impact on the community structure [3]. However, different microbiome communities could be correlated with field management. Soil bacterial abundance and diversity also vary substantially under different soil physicochemical properties [4]. Further experimental are required to better define the correlation between soil physicochemical properties and microbial diversity to BSR disease Figs. 2–4.

## 2 Experimental design, materials, and methods

### 2.1 Sites Selection

Study was conducted in three plantation sites in Sabah, Malaysia. The estates are located at Sandakan (Kam Cheong Plantation), Lahad Datu (FGV Ladang Sahabat) and Tawau (Warisan Gagah). Each estates incorporate different disease management and agronomic practices. Kam Cheong Plantation applied organic acids, FGV Ladang Sahabat carried out sanitation and Warisan Gagah applied microbial products in managing BSR disease. A total of 50 soil samples were randomly collected from the surrounding palms which represents 0.01% of the total palms in the study area. Three plots which are disease free, high and low BSR disease were collected from each estates. The chosen plots are within 30–40 hectare and the palm density is 136 tree per hectare. Sampling in each plots were separated by a minimum of 10 metre. BSR incidences data in the study area was provided by the participating estates.

### 2.2 Soil sampling and DNA extraction

Soil samples were collected at three different zones around the palm trees to represent spatial heterogeneity. The three zones are harvest path, circle and windrow. Soil in the sampling plot were taken using an auger (15 cm in depth), kept in zip-locked plastic bag and transported to laboratory. For each plot, soil samples collected from harvest path, circle and windrow were thoroughly mixed to obtain a single representative. Samples were air dried for five hours, grinded using mortar and pestle and sieved through 2 mm mesh and preserved temporarily at −20 ˚C. Soil DNA was extracted using PowerSoil® DNA Isolation Kit (MoBio Laboratory, CA, USA). The extracted DNA was checked using 0.8% w/v agarose gel electrophoresis and quantified using NanoDrop 2000 (Thermo Scientific).

### 2.3 Library preparation and next generation sequencing

Library prepration and 16S Metagenomics sequencing was performed at BioEasy Sdn. Bhd. DNA was amplified using primers desgined to target V3 and V4 regions. Forward Primer = 5′-TCGTCGGCAGCGTCAGATGTGTATAAGAGACAGCCTACGGGNGGCWGCAG-3′, Reverse Primer = 5′-GTCTCGTGGGCTCGGAGATGTGTATAAGAGACAGGACTACHVGGGTATCTAATCC-3′. PCR amplification template out of a DNA sample using region of interest‐specific primers with overhang adapters attached. AMPure XP beads was used to purify the 16S V3 and V4 amplicon away from free primers and primer dimer species. Dual indices and Illumina sequencing adapters were attached using the Nextera XT Index Kit. A second PCR was performed to clean up the final library before quantification. Libraries were quantified using the Qubit™ dsDNA HS Assay Kit (Thermo Fisher Scientific), normalized, pooled, and paired-end sequenced using the MiSeq Illumina Platform.

### 2.4 Statistical analysis

Upon FASTQ screening for adapter contamination using PEAT v1.2, FASTQ reads were subjected to BBMerge: A paired-end read merger v7.3. Reads obtained after quality control were subjected to taxonomic analysis against reference database (Greengenes 13_8). Matches read is assigned to the Operational Taxonomic Unit (OTU) defined by that reference sequence. Alpha diversity (Chao1, observed OTUs, Shannon) was generated using Qiime alpha_rarefraction script meanwhile beta diversity (PCoA) UniFrac method was generated using beta_diversity script in Qiime version 1.8.

## Declaration of Competing Interest

The authors declare that they have no known competing financial interests or personal relationships which have, or could be perceived to have, influenced the work reported in this article.

## Appendix Supplementary materials

Image, application 1
